# Fermentation dynamics of millet beverages: Microbial interactions, nutritional enhancements, and health implications

**DOI:** 10.1016/j.fochx.2025.102199

**Published:** 2025-01-19

**Authors:** Tanu Tomar, Angel Sachdeva, Joydeep Dutta, Abdel Rahman Mohammad Al Tawaha, Arun Karnwal, Tabarak Malik, Manickam Selvaraj

**Affiliations:** a Department of Microbiology, School of Bioengineering and Biosciences, Lovely Professional University, Punjab 144411, India; b Department of Zoology, School of Bioengineering and Biosciences, Lovely Professional University, Punjab 144411, India; c Department of Biological Sciences, Al Hussein bin Talal University, PO Box 20, Maan, Jordan; d Department of Microbiology, Graphic Era (Deemed to be University), Dehradun 248009, Uttarakhand, India; e Department of Biomedical Sciences, Institute of Health, Jimma University, Ethiopia; f Department of Chemistry, Faculty of Science, King Khalid University, Abha*, 61413,* Saudi Arabia; g Research Centre for Advanced Materials Science (RCAMS), King Khalid University, PO Box 9004, Abha *61413,* Saudi Arabia

**Keywords:** Millets, Fermentation, Beverages production, Bioactive compound, Microbial dynamics, Nutritional qualities

## Abstract

Fermented millet beverages are gaining attention as a sustainable and nutritious alternative to traditional functional foods, combining the nutritional benefits of millets with the transformative effects of fermentation. This review explores the microbial dynamics, biochemical changes, and health benefits of these beverages. Fermentation boosts nutrient bioavailability, reduces anti-nutritional factors, and produces bioactive compounds like antioxidants and probiotics that support gut health, metabolism, and immunity. It also enhances the synthesis of vitamins, minerals, and peptides, offering potential benefits for managing chronic conditions. Key factors such as temperature, pH, oxygen levels, and substrate composition influence fermentation, with specific microorganisms enhancing both nutritional and sensory qualities. These beverages align with sustainability goals, as millets thrive in resource-limited environments, and their gluten-free nature caters to dietary needs, including those with celiac disease. The review highlights the cultural significance of millet beverages while advocating for their integration into modern health markets and commercial viability

## Highlights

• Fermented millet beverages boost nutrition by enhancing vitamins, minerals, probiotics.
• Fermentation improves millet's digestibility, reduces anti-nutrients, adds bioactives.
• Millet fermentation uses microbes to create gut health and cognitive-promoting drinks.
• Advances in microbial ecology and strain improvement support innovative millet products.
• Fermented millet beverages offer dietary diversity, sustainability, and functional innovation.

## 1 Introduction

In recent years, fermented millet beverages have gained attention as culturally significant and nutritionally potent alternatives to conventional functional foods. These beverages leverage the inherent richness of millet—naturally high in fibre, vitamins, and minerals—while incorporating the transformative power of fermentation. This process enhances nutrient bioavailability, improves digestibility, and introduces probiotics, positioning these beverages as comprehensive solutions for gut health and overall wellness. Millets, often referred to as “Nutri-cereals,” are resilient cereal crops that feed nearly 30 % of the global population and rank sixth in worldwide cereal production (Singh, Rasane, Nanda, Ercisli, & Verma, 2024). They are rich in bioactive compounds such as beta-glucan, phenolic acids, flavonoids, and carotenoids (Sunil, Gowda, Nayak, & Rawson, 2024). Compared to wheat and rice, millets offer three to five times the nutrient density, making them a superior choice for addressing dietary deficiencies, particularly in regions with limited food diversity.

**Fig. 1:**
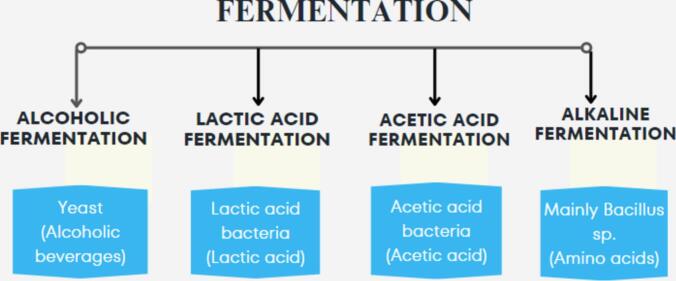
Types of fermentation.

There are various types of fermentation, classified based on the end products produced and the microorganisms involved, as shown in Fig. 1. Fermentation amplifies millet's nutritional profile by increasing the bioavailability of essential minerals, reducing anti-nutritional factors, and generating bioactive compounds like antioxidants and antimicrobial agents. This enzymatic process, often facilitated by Lactobacilli, promotes the synthesis of B-complex vitamins and breaks down complex plant structures, releasing trapped nutrients (Potter & Hotchkiss, 2012). The resulting beverages combat inflammation, improve digestive health, and may offer cognitive and immune benefits (Panigrahi, Nanda, Sagar, & Neetu Singh, 2024). Beyond individual health, fermented millet beverages align with global sustainability goals. Millets thrive in harsh climates and require minimal water, making them an ideal crop for regions grappling with food security challenges. Developing value-added products like fermented beverages not only enhances dietary diversity but also supports local economies (Tamang et al., 2020).

The cultural significance of fermented millet beverages is deeply rooted in the culinary traditions of Asia and Africa, where millet is a staple food. Drinks like “Masvusvu” from Zimbabwe, “Kunu-zaki” from Nigeria, and “Mangisi” reflect this heritage while offering innovative pathways for addressing modern health concerns (Kaur, Shekhar, & Prasad, 2024). By blending traditional techniques with advancements in nutritional science, these beverages bridge the gap between heritage and contemporary dietary demands.

This review explores the transformative impact of fermentation on millet's nutritional properties and identifies key microbial strains responsible for these changes. It highlights the role of fermentation in enhancing nutrient bioavailability and mitigating lifestyle-related health issues (Sunil et al., 2024). Additionally, it identifies gaps in the long-term health implications of fermented millet beverages and their role in immune support (Mensah et al., 2024). By examining the production processes, microbial dynamics, and health benefits, this review underscores the potential of fermented millet beverages as versatile, sustainable, and health-promoting dietary options. These beverages represent a critical addition to modern diets, advancing nutrition, sustainability, and the global appreciation of millets' remarkable potential.

## 2 Bioactive compounds and properties of millets

Plants offer various secondary metabolites, with phenolics, particularly flavonoids, standing out as a significant group. These secondary metabolites encompass a range of bioactive compounds that exert considerable effects on various biological systems. Consuming foods abundant in bioactive compounds, especially whole grains, fruits, and vegetables, enhances nutritional value and offers significant health benefits (Sunil et al., 2024). These bioactive compounds may aid in diminishing inflammation, decreasing oxidative stress, and alleviating metabolic challenges, mainly through their influence on energy regulation and utilization (Shrestha et al., 2024).

Bioactive substances can be grouped into three primary categories: (a) phenolic compounds, (b) alkaloids, and (c) terpenes and terpenoids. Their synthesis occurs through four main biochemical pathways: (1) the mevalonic acid pathway, (2) the non-mevalonate pathway (MEP), (3) the shikimic acid pathway, and (4) the malonic acid pathway (Liu et al., 2023). Alkaloids primarily arise from aromatic amino acids via the shikimic acid pathway, while aliphatic amino acids are derived from the tricarboxylic acid cycle. Phenolic compounds originate from the shikimic and malonic acid pathways, whereas terpenes are produced via the mevalonic acid and non-mevalonate pathways (Panigrahi et al., 2024). The phytochemicals exhibit diverse biological effects, including antioxidant activity, antibacterial properties, modulation of the immune system, regulation of detoxification enzymes, anticancer effects, inhibition of platelet aggregation, and alterations in hormone metabolism. The therapeutic properties of these bioactive compounds underscore their importance in enhancing human health and fostering the development of functional food items.

**Phenolic compounds,** an essential group of phytochemicals found in millets, serve as critical dietary antioxidants. The presence of these compounds, defined by their aromatic ring and hydroxyl groups, is crucial for human nutrition (Balli et al., 2023). Brown finger millet, for example, has a notably more significant amount of polyphenols (0.1 %) than white millet (0.003 %). Malted millet flour has about 38.36 mg of total phenols per 100 g, whereas finger millet varies between 265 and 373.15 mg/100 g of total phenolics. The methanolic extracts of Kodo millet grains exhibit elevated levels of antioxidant activity and phenolic content (Kumari, Bhinder, Singh, & Kaur, 2023). Plants contain various phenolic compounds, such as flavonoids, phenolic acids, lignans, and phytoestrogens, with phenolic acids and flavonoids being the most significant components in our diet.

**Hydroxycinnamic acids,** a group of phenolic compounds, are abundant in millets and contribute to their health benefits. These compounds include ferulic acid, caffeic acid, p-coumaric acid, and sinapic acid, known for their antioxidant, anti-inflammatory, and anticancer properties (Pujari & Hoskeri, 2022). Millets such as pearl millet (*Pennisetum glaucum*), finger millet (*Eleusine coracana*), and foxtail millet (*Setaria italica*) contain significant amounts of hydroxycinnamic acids, especially ferulic acid, which is the most prevalent. These compounds help reduce oxidative stress, support heart health, and prevent chronic diseases like diabetes and cancer. Additionally, hydroxycinnamic acids in millets enhance the bioavailability of essential nutrients and protect against microbial infections (Xiang et al., 2023). The antioxidant properties of these acids are beneficial in preventing lipid peroxidation and improving overall health. With their diverse health-promoting effects, millets rich in hydroxycinnamic acids serve as a functional food that promotes wellness.

**Sinapic acid**, a bioactive phenolic compound, possesses significant antioxidant, anti-inflammatory, anticancer, anti-mutagenic, neuroprotective, and antibacterial properties (Sunil et al., 2024). Inflammation plays a key role in chronic diseases, with Nrf2 acting as a regulator of anti-inflammatory responses by activating gene expression through the antioxidant response element (ARE) (Saha, Buttari, Panieri, Profumo, & Saso, 2020). Nrf2 signaling inhibits mediators like cyclooxygenase (COX-2) and inducible nitric oxide synthase (iNOS), which are critical for reducing inflammation and preventing conditions such as arthritis, epithelial neoplasms, and musculoskeletal disorders.

**p-coumaric acid,** a phenolic compound prevalent in millets, contributes significantly to its antioxidant properties. This compound exhibits significant anti-inflammatory, anticancer, and antioxidant properties while reducing LDL cholesterol oxidation and lipid peroxidation (Singh et al., 2022). Studies have identified soluble and insoluble-bound forms of p-coumaric acid in various millet varieties, with the bound fractions often containing higher concentrations (Gao et al., 2024; Shahidi & Hossain, 2023). For instance, barnyard millet is notably rich in p-coumaric acid, which has been shown to inhibit the formation of advanced glycation end-products, thereby offering protective effects against glycoxidation-induced protein conformational changes (Srivastava et al., 2021). Processing methods can influence the p-coumaric acid content in millets. Fermentation, for example, has been found to significantly increase p-coumaric acid levels in pearl millet, enhancing its antioxidant capacity (Purewal et al., 2020). Incorporating millets such as barnyard and pearl millet into the diet can provide health benefits attributed to their p-coumaric acid content, including antioxidant activity and potential protection against oxidative stress-related conditions. In millets, p-coumaric acid is present in varying amounts: finger, foxtail, and Little millet contain 1.81–41.1, 3.66–196.62, and 2.71–70.36 μg/g p-coumaric acid, respectively. Kodo millet contains 1.38 ppm of the compound.

**Ferulic acids,** a notable phenolic compound, is richly present in millets, providing considerable health advantages thanks to its potent antioxidant, anti-inflammatory, and antimicrobial characteristics. Millets like finger millet (Ragi), pearl millet (Bajra), foxtail millet, and proso millet are abundant in ferulic acid, which is mainly found in the outer layers of the grain, such as the bran and pericarp (Shrestha et al., 2024). Ferulic acid improves the beneficial characteristics of millets by neutralizing free radicals, which helps to diminish oxidative stress and decrease the likelihood of chronic conditions such as heart disease, diabetes, and cancer (Singh et al., 2022). For example, pearl millet is rich in ferulic acid, which plays a role in its ability to lower blood sugar and provide anti-diabetic benefits. Similarly, finger millet is celebrated for its ability to protect the nervous system, thanks to its rich ferulic acid content (Martinez-Lopez et al., 2021).

Adding millet-based foods abundant in ferulic acid to everyday meals promotes health and wellness, highlighting their significance in advancing functional food and nutraceutical innovations (Tanwar, Panghal, Chaudhary, Kumari, & Chhikara, 2023). The levels of ferulic acid in millets differ significantly, i.e., 41–405.0, 47–988.78, 54.65–254.20, 29.94–133.58, 27.88–1445.06 μg/g free or bound ferulic acid in finger, pearl, foxtail, little, and Kodo millets respectively (Sharma et al., 2023; Patel et al., 2024). Research indicates that ferulic acid offers protective advantages for the kidneys by improving blood sugar management and lowering oxidative stress via alterations in kidney structure (Sharma et al., 2023).

**Hydroxybenzoic acids,** Benzoic acid is the precursor to hydroxybenzoic acids, which comprise 70–71 % of the phenolic acids found in grains. Prominent hydroxybenzoic acids encompass protocatechuic, p-hydroxybenzoic, vanillic, gallic, gentisic, and syringic acids, with protocatechuic acid standing out as the leading free phenolic (Yuan et al., 2022). The concentration of hydroxybenzoic acid in finger millet is around 45 mg per 100 g. Little millet boasts elevated levels of benzoic acid (45.56 mg/g) and is enriched with p-hydroxybenzoic and vanillic acids, which provide a range of benefits, including antioxidant, anti-inflammatory, antibacterial, and hepatoprotective effects (Babypriyanka et al., 2024).

**Syringic acids,** are bioactive phenolic compounds in various plant-based foods, such as millet, and are recognized for their antioxidant, anti-inflammatory, and antimicrobial characteristics. This compound is classified as a hydroxybenzoic acid and is usually obtained through the phenolic acid biosynthesis pathway. Millets, including pearl millet (*Pennisetum glaucum*), finger millet (*Eleusine coracana*), and foxtail millet (*Setaria italica*), are abundant in phenolic acids like syringic acid, which play a significant role in their health advantages (Zhang et al., 2021). Studies show that millet's syringic acid is a powerful antioxidant, effectively neutralizing free radicals and lowering oxidative stress (Shimsa, Soumya, Mondal, & Mini, 2023). This is essential for reducing the risk of long-term health issues such as heart disease, diabetes, and cancer. Moreover, syringic acid has demonstrated promising anti-inflammatory properties, aiding in regulating the inflammatory response in ailments like arthritis and gastrointestinal issues. In research focused on pearl millet, it was discovered that the grain possesses a notable level of syringic acid, which plays a key role in its elevated antioxidant activity in conjunction with various phenolic compounds. Similarly, finger millet, widely enjoyed in different preparations across Africa and Asia, is rich in syringic acid, contributing to its overall nutritional profile. Goudar et al. (2023) documented diverse concentrations in millets: 2.44, 30, 8.59, 21.56, 28.22, and 15.72 μg/g in pearl, finger, foxtail, barnyard, kodo, and proso millet, respectively.

**Flavonoids,** a varied group of plant-derived phenolic compounds, demonstrate significant bioactive properties, including anticancer, anti-diabetic, anti-inflammatory, neuroprotective, and cardio-protective effects. These compounds are classified according to the saturation level of the central heterocyclic ring, distinguishing between unsaturated varieties (such as anthocyanidins, flavones, and flavonols) and saturated varieties (including flavanones and dihydroflavonols) (Martinez-Lopez et al., 2021). Millets provide abundant bioactive flavonoids, such as quercetin, kaempferol, myricetin, apigenin, catechin, naringenin, and daidzein. Catechin, abundant within millet seed coats, is linked to properties that combat cancer, diabetes, and aging (Tang et al., 2022). Kodo millet has a catechin content of 1.10 ppm, providing anti-obesity advantages, whereas foxtail millet boasts significant amounts of catechin (31.65 μg/g bound) and apigenin (125.16 μg/g bound), associated with anti-diabetic and anticancer properties (Ofosu et al., 2020). Finger millet boasts a high concentration of quercetin, reaching up to 2100 μg/g in its soluble form. Little millet, with its elevated kaempferol content, reduces the risk of chronic diseases. The total flavonoid content differs across various types of millets: foxtail millet exhibits 244.34 μg/g (soluble) and 368.28 μg/g (bound), whereas little millet shows 108.97 μg/g (soluble) and 323.23 μg/g (bound). Malted pearl millet flour provides 34.73 mg/100 g of flavonoids (Tang et al., 2022).

Table 1 highlights the key bioactive compounds present in millets, including their chemical components, associated health benefits, and how fermentation impacts their bioavailability and efficacy. The data reflects recent studies on the potential of millet-based bioactive substances in promoting health.

**Table 1:** Bioactive Compounds in Millets: Classes, Key Components, Health Benefits, and Effects of Fermentation.

**Bioactive Compound Class**	**Key Components**	**Health Benefits**	**Effect of Fermentation**	**References**
Beta-glucans	(1, 3) (1,4)-β-D-glucans	Immunomodulation, Cholesterol reduction, Blood glucose regulation	Increased solubility and bioavailability	(Sunil et al., 2024).
Flavonoids	Quercetin, Apigenin, Luteolin	Antioxidant activity, Anti-inflammatory effects, Cardiovascular protection	Enhanced extraction and conversion to more bioactive forms	Martinez-Lopez et al., 2021
Phenolic Acids	Ferulic acid, p-Coumaric, acid, Caffeic acid	Antioxidant properties, Neuroprotection, Anti-aging effects	Increased free phenolic content and bioavailability	Singh et al., 2022
Feraxans	Ferulic-rich arabinoxylans	Prebiotic effects, Metabolic regulation, Gut health support	Structural modification leading to enhanced bioactivity	Shrestha et al., 2024
Lignins	Syringyl lignin, Guaiacyl lignin	Anticancer potential, Prebiotic effects, Antioxidant activity	Partial breakdown improving bioavailability	Tanwar et al., 2023
Carotenoids	Lutein Zeaxanthin β-carotene	Eye health Antioxidant protection Immune system support	Improved extraction and stability	Sharma et al., 2023

## 3 Key millet varieties for fermented beverage production

**Fig. 2:**
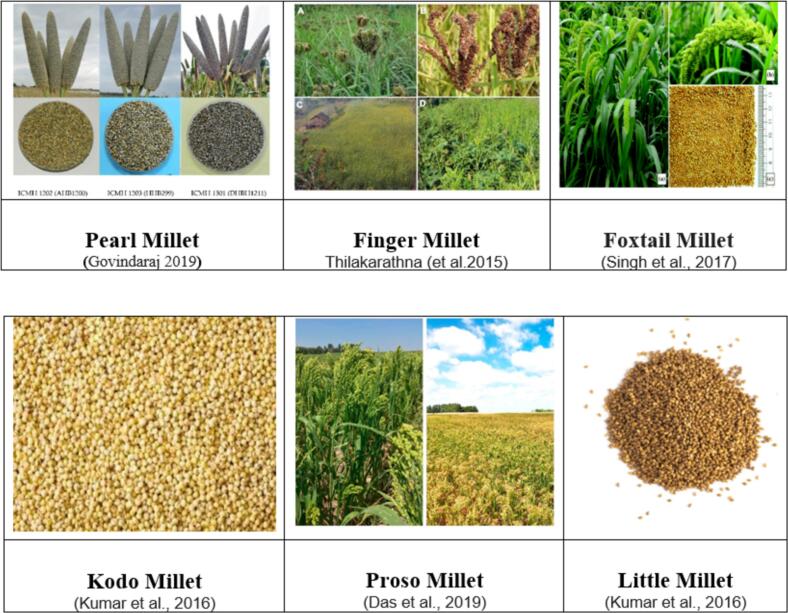
Millet Varieties Used in Fermented Beverages.

Millet varieties significantly influence the production of fermented beverages, as each variety brings its own unique characteristics that affect the final product's quality, nutritional profile, and sensory appeal. Among the most notable millet varieties used in fermentation are finger millet (*Eleusine coracana*), pearl millet (*Pennisetum glaucum*), foxtail millet (*Setaria italica*), barnyard millet (*Echinochloa crus-galli*), and proso millet (*Panicum miliaceum*). ‘foxtail millet’ (*Setaria italica*), ‘finger millet’ (*Eleusine coracana*), ‘proso millet’ (*Panicum miliaceum*), ‘kodo millet’ (*Paspalum scrobiculatum*), ‘barnyard millet’ (*Echinochloa* spp.), and ‘little millet’ *(Panicum sumatrense)* as shown in Fig. 2 (Sunil et al., 2024). These varieties, with their distinct nutritional and functional profiles, offer diverse benefits that are harnessed during the fermentation process.

**Finger millet** is particularly valued for its high calcium and iron content, which enhances the mineral composition of fermented beverages. The slight nuttiness of finger millet also contributes to a richer, fuller flavor profile, making it a popular choice for enhancing the taste of millet-based drinks. In addition, finger millet is often used in traditional Indian drinks like “ragi” beverages, known for their energy-boosting and health-promoting properties (Taylor et al., 2019).

**Pearl millet**, with its high fibre content and essential amino acids, is an excellent choice for improving digestive health. This millet variety imparts a more substantial mouthfeel to the fermented drink, making it more satisfying and beneficial for those seeking digestive support. In many African countries like Senegal and Nigeria, pearl millet is used to brew traditional beverages such as “bajra” and “kunu.” These beverages are not only consumed for their refreshing qualities but also hold cultural and social significance, often being served at community gatherings and ceremonies.

**Foxtail millet**, known for its antioxidant properties and low glycemic index, is favored by health-conscious consumers. It is often used in fermented beverages aimed at aiding blood sugar management. The health benefits of foxtail millet, combined with its pleasant flavor and texture, make it a desirable option for functional fermented drinks.

**Barnyard millet** is another variety that is gaining popularity due to its high fibre content and ability to aid in digestion. It is used in various fermented products, providing both nutritional value and a light, refreshing taste.

**Proso millet**, often used to make porridge and fermented beverages such as Chinese yellow wine, offers unique flavors due to its distinct profile. Though its role in influencing flavor and microbial communities during fermentation remains somewhat unclear, its use in fermentation is valued for adding complexity to the final product (Zhao & Gao, 2024). Proso millet is also a staple in some parts of Asia and is increasingly being recognized for its potential in various food applications, including fermentation.

The geographical distribution of these millet varieties significantly shapes their cultural importance and the traditional practices associated with their use in fermented beverages. For instance, **pearl millet** is widely used in Africa, especially in countries like Senegal and Nigeria, where it plays an integral role in brewing traditional beverages like “bajra” and “kunu.” These beverages are often prepared for social events, religious ceremonies, and community celebrations, underscoring the cultural value of millet in these societies. Similarly, in **India**, particularly in the South, **finger millet** is commonly used to make “ragi” drinks, which are celebrated for their health benefits, including boosting energy and supporting overall wellness (Taylor et al., 2019).

The methods of preparing millet-based fermented beverages vary widely across different regions, reflecting local customs and culinary traditions. In many cases, traditional fermentation involves spontaneous fermentation using wild yeast and lactic acid bacteria. This results in beverages with complex flavors, varying alcohol content, and beneficial probiotic effects, highlighting the ingenuity of local communities in utilizing natural resources and preserving traditional food practices.

In South India, during the early historical period (around 300 BCE to 500 CE), millet played a central role in the diet, as documented in Sangam literature. This period, characterized by a village-based economy and a division of labor focused on activities such as hunting, gathering, fishing, agriculture, and pastoralism, saw millet and pulses as the dominant staples. These grains were often incorporated into traditional medicinal practices, reflecting a deep understanding of nutrition and health (Lepcha, Patra, & Saha, 2023).

Proso millet also has a long history of use in China, particularly in the production of yellow wine. While its precise role in fermentation is still under investigation, it is known to contribute distinct flavors to the beverage and influence microbial communities during fermentation. Its potential as a functional ingredient in fermentation continues to be explored, especially in terms of its impact on flavor and microbial diversity (Zhao & Gao, 2024). In Zimbabwe, finger millet is commonly used in the preparation of composite fermented beverages, where it is mixed with other ingredients such as skim milk. Research has focused on optimizing microbial cultures to maximize the quality and nutritional benefits of these drinks. Finger millet-based fermented beverages are popular and widely consumed in Zimbabwe as a healthful, traditional cuisine (Dhliwayo, Nyanga, Chopera, Matsungo, & Chidewe, 2022).

Moreover, in the **Eastern Himalayas**, fermented drinks made from **finger millet**, such as “kodo ko jaanr,” are traditionally enjoyed. These beverages are moderately alcoholic and are a staple in the region, offering both nutritional and cultural value. Overall, the diversity of millet varieties, their nutritional advantages, and the traditional knowledge surrounding their use in fermented beverages showcase the significance of millets in global culinary practices. The unique characteristics of each millet variety contribute not only to the flavor, texture, and nutritional content of fermented beverages but also to the cultural heritage and social practices tied to these traditional drinks. The fermentation of millets, particularly through local methods and with specific microbial cultures, continues to play a crucial role in enhancing the bioactive properties of these grains, offering health benefits that extend beyond simple nutrition.

In 2005, the researcher examined 40 cultures of kodo ko jaanr, which were gathered from Sikkim and Darjeeling highlands in India. They underwent analytical and microbiological investigations. The lactic acid bacteria and yeast population was found to be 7.1 and 5.9 log cfu g1, respectively. The yeasts included ‘*Saccharomyces cerevisiae’*, ‘*Candida glabrata’*, ‘*Saccharomycopsis fibuligera’*, ‘*Pichia anomala’*, and ‘*Lactobacillus bifermentans’* in the sample of ‘Kodo Ko jaanr’, while LAB included *Pediococcus pentosaceus*. Pathogenic pollutants were checked for in the samples. No sample included any indication of ‘*Enterobacteriaceae’, ‘Bacillus cereus’, or ‘Staphylococcus aureus’*. 4.1, 69.7 %, 0.27 %, and 4.8 % were product, moisture content, pH, acidity, and alcohol concentration. The crude fibre content of ‘Kodo ko jaanr’ is high. Like substrate, the amount of ash, fat, protein, and calories did not change (Gomathy et al., 2023). White finger millet is a significant source of vitamins and nutrients Table 1. According to the study's findings, it serves as an excellent carrier matrix for development and could be a suitable option for individuals who are lactose intolerant or strictly vegan (Navyashree, Buvaneswaran, Sunil, Rawson, & Natarajan, 2023). Table 2 provides an overview of different millet varieties used in the production of fermented beverages across various regions. It covers the types of fermentation used (alcoholic and lactic acid), the typical sensory properties of these beverages, and the millet varieties that are most commonly employed in these processes.

**Table 2:** Fermented Millet Beverages: Varieties, Regions, and Types of Fermentation.

**Millet Variety**	**Region**	**Fermented Beverage**	**Type of Fermentation**	**Properties**	**Key component**	**Ref.**
Pearl Millet *(Pennisetum glaucum)*	Africa, India, Pakistan	Pito, Burukutu, Dolo, Tchapalo, Ikigage, Mangisi, Rabadi	Alcoholic & Non-Alcoholic (Lactic Acid)	Strong, sour, porridge-like consistency	Most widely used millet for beverages	Owheruo, Ifesan, & Kolawole, 2019; Badejo, Nwachukwu, Ayo-Omogie, & Fasuhanmi, 2020; Rani, Dabur, Bishnoi, & Jairath, 2024
Finger Millet *(Eleusine coracana)*	Africa, India	Mahewu, Uji, Murrah, Handi, Khamb, Kinkeliba	Non-Alcoholic (Lactic Acid)	Thin, tart, slightly sweet	Highly nutritious, good source of calcium and	Navyashree et al., 2023; Patil, Singh, & Patel, 2023
Foxtail Millet *(Setaria italica)*	China, India, Africa	Handi, Kodo, Murria	Non-Alcoholic (Lactic Acid)	Milky, sweet, slightly alcoholic	Easily digestible, gluten-free	Pan et al., 2019; Chaturvedi, Kumar, Pratap, & Singh, 2024
Kodo Millet *(Paspalum scrobiculatum)*	India, Africa	Handi, Kodo, Murria	Non-Alcoholic (Lactic Acid)	Thin, slightly sweet, porridge-like	Rich in dietary fibre, easily digestible	Sharma & Sharma, 2021; Dey, Saxena, Kumar, Maity, & Tarafdar, 2022; Dey, Saxena, Kumar, Maity, & Tarafdar, 2024
Proso Millet (*Panicum miliaceum*)	India, China, Central Asia	Bor, Handi, Idiyappam	Non-Alcoholic (Lactic Acid)	Creamy, slightly sweet, mild flavor	High in protein and minerals, gluten-free	Sharma & Sharma, 2021; Mathanghi, Kanchana, & Perasiriyan, 2020; Mathanghi, Perasiriyn, & Karthiayani, 2021

## 4 Microbial dynamics and nutritional enhancements in millet fermentation

Fermented foods are considered to be higher in nutrients than their unfermented alternatives. Fermented foods have more nutritional value because they include microorganisms involved in the fermentation process. There are three methods by which these microorganisms ferment food:1) Microorganisms create complex vitamins and other growth factors, break down intricate molecules, and are both catabolic and anabolic. 2) Inedible compounds release nutrients trapped in the structures and cells of plants. This phenomenon is particularly evident with individual seeds and grains. 3) An alternative approach to augmenting the nutritional qualities of plant material involves the enzymatic breakdown of non-digestible polymers into simple carbohydrates and their byproducts, such as hemicelluloses, cellulose, and related polymers. Microbial enzymes can improve the cellulose-containing compounds in food that are fermented to be suitable for human consumption (Sharma, Garg, Kumar, Bhatia, & Kulshrestha, 2020).

The fermentation method permits the production of vitamins and growth factors for a healthy diet and the breakdown of complicated substances. Reduced anti-nutrient agents occur throughout lactic acid fermentation, although proteins and amino acids become more soluble. By decreasing the indigestible components of plants, including cellulose, hemicelluloses, and polygalacturonic and glucuronic acids, fermentation raises the availability of minerals and trace elements (Table 2). However, the length of fermentation and the ingredients' makeup can impact the finished product's nutritional value (Kitessa, Bacha, Tola, & Murimi, 2024).

In 2023, The study examined the composition of four millet varieties, focusing on phenolic compounds and starch content. Nigerian pearl millet (PMN) exhibited the highest free phenols at 0.74 mg/g; total phenolic content was greater with acidic extraction methods. Resistant starch (RS) ranged from 9.26 g/100 g in foxtail millet to 24.25 g/100 g in finger millet. Italian pearl millet (PMI) was then fermented using various microbial mixtures to produce FPM1, FPM2, and FPM3. Following fermentation, pH levels decreased while total titratable acidity (TTA) increased, with FPM3 showing the lowest pH at 3.59 and FPM1 the highest TTA at 26.10 mL. Notably, FPM2 and FPM3 had higher total phenolic content than unfermented millet, with 22 % and 15 % increases, respectively. Fermentation also enhanced starch content, particularly slow-digestible starch (SDS). Regarding prebiotic activity, both FPM2 and FPM3 effectively promoted the growth of *Bifidobacterium breve* B632, with FPM2 demonstrating superior support at 48 and 72 h. These findings highlight the nutritional benefits of fermented millet as a substrate for beneficial bacteria (Balli et al., 2023).

In 2024, a study examined how proso millet influences the microbial communities and volatile flavor compounds in proso millet Baijiu, a new variant of Shanxi light-flavoured Baijiu, and explored the relationship between these factors. The introduction of proso millet shifted the primary flavor compounds from ethyl acetate, ethyl hexanoate, and butyl acetate to compounds like oct-2-ene, 2-butanol, and 4-methylpentanal. Proso millet significantly altered the microbial composition during fermentation, especially in the first 14 days. The formation of unique flavor compounds in proso millet Baijiu showed a strong positive correlation with fungi from the genera *Rhizopus, Papiliotrema, Wickerhamomyces, Aspergillus, and Thermoascus,* while showing a negative correlation with bacteria from *Weissella, and Acinetobacter*. They break down starches and produce various metabolites, such as esters and organic acids, to develop unique aroma compounds. This not only improves fermentation efficiency but also enriches the sensory profile of the final product. The low alcohol production rate observed in Fenjiu may be attributed to the abundance of fungi from the *Psathyrella* genus and certain bacteria. Overall, while proso millet enhanced the flavor profile of light-flavoured Baijiu by promoting specific microbial communities, it did not increase alcohol concentration. This research sets the groundwork for further studies to improve the flavor of light-flavoured Baijiu through changes in fermentation materials (Zhao & Gao, 2024).

In 2020, researchers showed that drinks made with coarse grains and millets had an apparent high viscosity, suggesting that they would be satiating and contain energy. The flavouring components influenced the three beverages' sensory attributes, color, and texture. Enhancing drinks is an ideal means to get minerals and energy. These beverages have strong phenolic and radical scavenging properties and are nutritious. When such drinks can be taken as a snack or supplement between meals, they can deliver calories and other necessary elements, which can help the elderly's nutritional state. (Bembem and Murugkar 2020). The following study has also demonstrated the value of Mahewu for feeding infants and young children and consumers' favourable perceptions of its nutritional attributes. Fermentation is additionally referred to as activating phytases, which increase the bioaccessibility of minerals and proteins by catalyzing the degradation of phytic acid into a form with low affinity for proteins. Therefore, there is potential to improve Mahewu into a beverage with even more health-promoting properties by carefully balancing the raw elements and processing techniques. The improved product should ideally have a low glycaemic index, be high in micronutrients, be low in phytic acid, be high in essential amino acids, be rich in antioxidants, and include climate-smart cereal ingredients. (Kudita, Schoustra, Mubaiwa, Smid, & Linnemann, 2023).

**Fig. 3:**
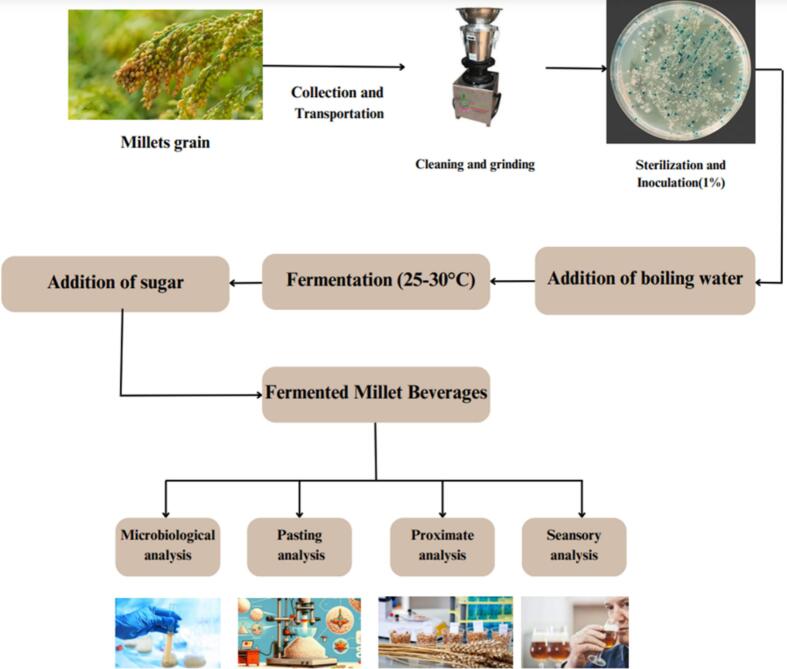
Production of fermented Millets Beverages.

The two main factors determining a food's nutritional value are its digestibility and the amount of necessary nutrients it contains. The fermentation process can increase both digestibility and nutrition. The enzymes produced by the cultured microorganisms may first break down the macronutrients during the fermentation process, as shown in Fig. 3. Fermentation can enhance food's nutritional accessibility in several ways, including raising nutrient density and improving nutrient availability. The latter can be achieved by breaking down anti-nutritional factors, promoting absorption by producing promoters, pre-digesting certain meal elements, and modifying the mucosa's ability to absorb nutrients. (Sharma et al., 2020).

This study examined a variety of basic physicochemical parameters, microorganism profiles (with 284 and 15 genera of ‘bacteria’ and ‘fungus’, respectively, found during millet Huangjiu fermentation), and 95 flavor compounds (which includes ‘31 esters’, ‘23 alcohols’, ‘13 alkanes’, ‘7 ketones’, ‘6 acids’, ‘3 phenols’, ‘2 aldehydes’, and ten additional categories of volatile substances). Correlations between these data were also established and examined. Bacillus was the most common at the genus level, followed by Paenibacillus and Weissella. During millet Huangjiu fermentation, 537 connections between flavor components and microorganisms have been identified. *P* < 0.05 indicated that 153 bacteria were necessary for synthesizing the major flavor components. *Psychrobacter, Anoxybacillus, Chryseobacterium, and Sporolactobacillus* were the top five dominating genera of microorganisms that produced flavor (Yan et al., 2022).

Scientists (Navyashree et al., 2023) intend to produce the vegan White Finger Millet Probiotic Drink, improve the components used in the composition, and research the finished product's physiochemical and nutrient content characteristics. The optimal conditions for producing the probiotic beverage derived from White Finger Millet were ascertained by applying the ‘Box-Behnken design’.Optimization was achieved by examining the effects of the independent factors, such as ‘White Finger Millet flour’ (10–14 % *w*/*v*), ‘microbial inoculum’ (1–5 % *v*/v), and ‘sugar’ (5–15 % w/v), on the response factor, such as total solid content (TSS), pH, microbial count, and viscosity. According to the study, White Finger Millet is a superior carrier matrix for development and may substitute for lactose-sensitive or strictly vegan consumers.

The microbial enzymatic activity during fermentation creates a cascade of nutritional improvements. For instance, lactic acid bacteria produce vitamin B complexes, particularly B12 and folate, while simultaneously lowering the pH, which enhances mineral solubility and absorption. The proteolytic activity *of Bacillus species* increases protein digestibility by breaking down complex storage proteins into smaller peptides and free amino acids, resulting in improved protein quality scores. This is particularly evident in traditional beverages like Mahewu, where fermentation increases the lysine content, a limiting amino acid in cereal grains.

The metabolic activities of these microorganisms also contribute to the degradation of anti-nutritional factors. Fungal species, particularly those from the Aspergillus genus, produce enzymes that break down tannins and polyphenols, which typically interfere with protein digestion and mineral absorption. Simultaneously, the fermentation process enhances the synthesis of bioactive compounds: phenolic compounds are released from bound forms, increasing their antioxidant capacity, while certain strains of Lactobacillus contribute to the production of gamma-aminobutyric acid (GABA), a beneficial bioactive compound.

The duration and conditions of fermentation significantly impact these nutritional transformations. Research has shown that optimal fermentation periods of 24–48 h maximize the production of beneficial compounds while minimizing the formation of undesirable metabolites. Temperature and pH also play crucial roles in determining which microbial communities dominate and which enzymatic pathways are activated. For example, studies on proso millet Baijiu demonstrate that specific microbial populations early in fermentation (0–14 days) are critical for developing nutritional and sensory characteristics.

Modern analytical techniques have revealed the complex interplay between microbial species during fermentation. For instance, in millet Huangjiu fermentation, the identification of 284 bacterial and 15 fungal genera demonstrates the complexity of these microbial ecosystems. These diverse communities work in succession, with early colonizers creating conditions favourable for subsequent species, each contributing to the nutritional enhancement of the final product. Specific genera like Psychrobacter and Anoxybacillus have been directly correlated with the production of beneficial metabolites and the improvement of nutrient bioavailability.

Recent advances in fermentation optimization, such as the Box-Behnken design approach used in White Finger Millet probiotic beverage development, have enabled better control over these microbial dynamics. By carefully managing parameters such as inoculum concentration, substrate availability, and environmental conditions, it's possible to direct the fermentation process toward maximizing nutritional benefits while maintaining desirable sensory characteristics. This scientific approach has led to the development of functional beverages that serve as alternatives for specific dietary requirements and offer enhanced nutritional profiles compared to their unfermented counterparts.

The interplay between microbial dynamics and nutritional changes during the fermentation of millet beverages is crucial for enhancing their digestibility and nutritional value. Specific microbial communities, mainly lactic acid bacteria (LAB) and yeasts, are instrumental in producing various enzymes that target complex carbohydrates, proteins, and anti-nutrients. For instance, amylases produced by strains such as *Lactobacillus plantarum* can efficiently hydrolyze starches in millets into simpler sugars like glucose and maltose. This enzymatic breakdown increases the beverage's sweetness and makes carbohydrates more bioavailable, improving energy accessibility for consumers.

In addition to carbohydrates, the fermentation process involves significant protein degradation facilitated by proteolytic enzymes produced by microbial communities. For example, *Bacillus subtilis*, often used in millet fermentation, secretes enzymes that break down proteins into peptides and free amino acids, enhancing the amino acid profile of the final product. This is particularly beneficial as it increases the availability of essential amino acids critical for various bodily functions, including tissue repair and immune response.

Fermentation also plays a vital role in reducing the levels of anti-nutrients, such as phytic acid, which can bind essential minerals and hinder their absorption. Microbial phytases, particularly from species like Aspergillus oryzae, can degrade phytic acid during fermentation, thereby improving the bioavailability of minerals such as iron, zinc, and calcium. Studies have shown that the fermentation of finger millet can significantly reduce phytic acid content, thereby enhancing the absorption rates of these crucial minerals. For instance, fermented finger millet has been reported to have increased iron bioavailability, making it a more effective source of this essential nutrient for combating anaemia, particularly in at-risk populations.

Moreover, fermentation is known to boost the levels of specific vitamins, especially B vitamins. The metabolic activities of microorganisms during fermentation can lead to an increase in riboflavin (B2) and niacin (B3) levels. For example, research indicates that fermented pearl millet exhibits elevated niacin concentrations, contributing to its potential to improve dietary intake of this vital vitamin, which plays a significant role in energy metabolism and skin health. Table 3 presents the organic and inorganic nutrient content of various millet types, including proteins, fats, carbohydrates, fibre, and essential minerals like calcium, iron, magnesium, phosphorus, and potassium. These values provide insights into the nutritional benefits of millets as part of a balanced diet.

**Table 3:** Nutritional Composition of Millets: Organic and Inorganic Nutrients.

**Millet Type**	**Organic nutrients (g/100** **g)**				**Inorganic nutrients (mg/100** **g)**					**Ref.**
	**Protein**	**Fat**	**Carbohydrates**	**Fibre**	**Ca**	**Fe**	**Mg**	**P**	**K**	
**Finger Millet (Ragi)**	7.3	1.3	72.0	18.0	341	3.3	278	331	270	Jagati, Mahapatra, & Dash, 2021; Rathore, Singh, Kamble, Upadhyay, & Thangalakshmi, 2019
**Pearl Millet (Bajra)**	11.6	5.9	67.7	12.5	72	7.0	161	281	339	Sharma, Tripathy, Singh, Shah, & Chauhan, 2024
**Foxtail Millet (Kangni)**	7.9	3.9	66.7	8.4	31	2.4	172	282	222	Kumari et al., 2023; Verma, Joshi, Rana, & Bhatt, 2020
**Kodo Millet (Kodri)**	8.3	1.2	69.8	9.0	73	2.6	129	208	186	Shah, Dhir, Joshi, & Tripathy, 2023
**Proso Millet (Cheena)**	10.9	2.2	72.8	8.5	27	1.8	225	289	225	Rahman, Riad, Begum, Ali, & Saha, 2020; Das, Khound, Santra, & Santra, 2019
**Little Millet (Samai)**	9.7	1.9	74.1	8.6	348	2.8	179	230	209	Indirani, 2021; Dey et al., 2024
**Barnyard Millet (Swank)**	8.1	1.5	68.1	10.0	77	3.4	172	285	180	Kaur & Sharma, 2020; Kumari, Kajla, & Kaushik, 2021

## 5 Health benefits associated with fermented millet beverages

Fermented millet beverages offer intriguing health benefits, particularly concerning digestion, gut health, and blood sugar control. The fermentation process enhances the nutritional profile of millets by breaking down complex carbohydrates and proteins, which may improve digestibility. Studies have shown that fermentation increases the availability of bioactive compounds, such as amino acids and peptides, that can aid in gut health by promoting the growth of beneficial bacteria. For instance, lactic acid bacteria produced during fermentation can lower the pH of the gut environment, creating conditions that inhibit harmful pathogens and support the proliferation of probiotics. Research indicates these probiotics can enhance gut microbiome diversity, crucial for optimal digestion and overall gut health.

Regarding blood sugar control, fermented millet beverages may exert a positive influence through several mechanisms. Fermented products often have a lower glycemic index than their non-fermented counterparts, likely due to the breakdown of starches into simpler sugars during fermentation. This slower release of glucose into the bloodstream can help manage blood sugar levels more effectively. Some studies have reported that regular consumption of fermented millet products correlates with improved glycemic control in individuals with diabetes. However, results can vary; while some research demonstrates significant reductions in postprandial blood glucose levels, others have found minimal or no effects, suggesting that individual responses may differ based on factors such as dietary habits and metabolic health.

Despite these promising findings, gaps in the literature warrant further investigation. For example, while the benefits of fermented millet beverages on gut health and blood sugar management are acknowledged, the specific strains of microorganisms responsible for these effects are not well-defined across studies. Additionally, the long-term health impacts of regular consumption of these beverages remain underexplored. Further research is needed to establish standardized fermentation processes and identify which probiotic strains yield the most beneficial effects.

**Fig. 4:**
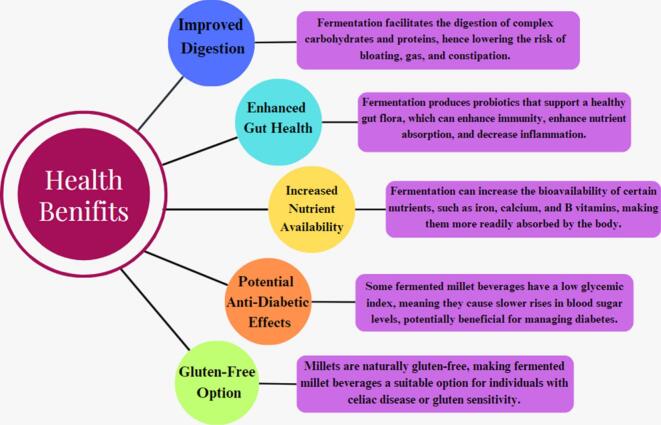
Health Benefits of Fermented Millet Beverages.

Millet is a type of tiny-seeded grain that has been farmed for millennia and is extensively used worldwide. Fermentation has traditionally been linked with several health benefits, and fermented foods have been a staple of diets in many cultures. (Sanlier, Gökcen, & Sezgin, 2019) There are various possible health benefits of millet consumption, as shown in Fig. 4 Their high fibre content facilitates better digestion and aids in blood sugar regulation. They can help control weight and are an excellent source of energy. Their numerous health benefits are making them more and more popular. The intake of fermented drinks may regulate the brain and central nervous system, which increases the gut microbiota (Fuloria et al., 2022).

Additionally, millet is a fantastic source of phytochemicals that are thought to decrease cholesterol, such as phytate and phytic acid. Some desirable chemopreventive compounds known as phytochemicals, including antioxidants in high concentrations in foods like millet, have been linked to these health advantages (Table 3). Because millet lacks gluten, it is one of the best options for individuals with celiac disease who are often annoyed by gluten in wheat and other more popular cereal grains. Furthermore, it helps those with diabetic and atherosclerosis. Millet extract taken from the coat has been shown to have a strong antibacterial and antifungal effect compared to whole wheat extract, perhaps because of its elevated polyphenol concentration in the seed coat (Majumder et al., 2024).

Patil et al. (2023) examine the characteristics of finger millet's dietary fibre and polyphenols and how they contribute to the grain's health advantages. They mentioned that It is a good source of calcium, iron, potassium, dietary fibres, and polyphenolic compounds. Its high iron content supports haemoglobin production in red blood cells. Being naturally gluten-free, ragi is suitable for individuals with gluten intolerance, which causes chronic inflammatory bowel disorders. Additionally, ragi offers significant health benefits, including antidiabetic, antioxidant, and antimicrobial properties, making it beneficial for both gluten-intolerant and diabetic individuals. In type 2 diabetic mice, it was observed that the protein content of ‘Korean foxtail millet’ and ‘proso millet’ markedly ‘increased plasma’, ‘adiponectin’, and ‘HDL cholesterol levels’ and produced substantial reductions in insulin levels in comparison to a casein diet. Additionally, proso millet increases both plasma levels and glycemic responses (Abhirami, 2024).

Fermented foods have garnered attention for their potential cognitive benefits, primarily attributed to their rich content of probiotics, bioactive compounds, and enhanced nutrient bioavailability. Probiotics, which are live beneficial bacteria found in fermented foods, can positively influence gut health, and emerging research suggests a strong connection between gut health and brain function, often referred to as the “gut-brain axis.” This connection implies that a balanced gut microbiome may enhance cognitive functions such as memory, attention, and mood regulation. Additionally, fermented foods are often rich in neurotransmitters, such as gamma-aminobutyric acid (GABA), which can have calming effects on the nervous system and may help reduce anxiety and stress. The fermentation process also increases the availability of vitamins, particularly B vitamins, which play crucial roles in brain health and function.

Moreover, antioxidants in many fermented foods can combat oxidative stress, contributing to cognitive decline. These factors suggest that incorporating fermented foods into one's diet may promote gut health and support cognitive well-being, highlighting their potential role in enhancing mental clarity, emotional stability, and overall cognitive performance. (Tanwar et al., 2023). Table 4 summarizes the potential health benefits associated with fermented millet products, such as improved digestion, enhanced gut health, increased nutrient bioavailability, and potential anti-diabetic effects. It includes evidence from research studies that highlight how fermentation can improve the health-promoting properties of millet-based foods and beverages.

**Table 4:** Health Benefits of Fermented Millet: Digestion, Gut Health, Nutrient Availability and other benefits.

**Benefit**	**Description**	**Evidence**	**Specific remark**	**Ref.**
**Improved Digestion:**	Fermentation breaks down complex carbohydrates and proteins, making them easier to digest and potentially reducing bloating, gas, and constipation.	Studies have shown that fermented millet products can improve digestive enzyme activity and gut motility (Shivakumar, Gunduraj, & Yanjarappa Sreenivasa, 2023)	It may be particularly beneficial for individuals with digestive issues.	Rizwana, Singh, Ahalya, & Mohanasundaram, 2023
**Enhanced Gut Health:**	Probiotics produced during fermentation contribute to a healthy gut microbiome, which can boost immunity, improve nutrient absorption, and reduce inflammation.	Research suggests that fermented millet beverages contain diverse probiotic strains with potential health benefits (Gebre et al., 2024)	may provide some relief from inflammatory bowel disease (IBD) and irritable bowel syndrome (IBS).	Paschapur et al., 2021
**Increased Nutrient Availability:**	Certain elements, like iron, calcium, and B vitamins, can become more bioavailable by fermentation, which facilitates the body's easier absorption of such nutrients.	Studies have demonstrated improved iron absorption from fermented millet compared to unfermented versions (Sabuz et al., 2023; Srivastava et al., 2021)	Can be particularly valuable for individuals with nutrient deficiencies.	Sabuz et al., 2023
**Potential Anti-Diabetic Effects:**	Some fermented millet beverages have a low glycemic index, meaning they cause slower rises in blood sugar levels, potentially beneficial for managing diabetes.	Research is ongoing, but some studies suggest a link between millet consumption and improved blood sugar control (Anitha et al., 2021; Ren et al., 2022)	More research is needed to confirm these findings.	Sabuz et al., 2023; Vedamanickam, Anandan, Bupesh, & Vasanth, 2020
**Gluten-Free Option:**	Since millet is inherently gluten-free, those who have celiac disease or gluten sensitivity may find fermented millet drinks to be an appropriate alternative.	This is a significant benefit for individuals who need to avoid gluten in their diet (Ren et al., 2022)	It helps those with diabetic heart disease and atherosclerosis	Rizwana et al., 2023; Paschapur et al., 2021
**Additional Benefits:**	Some studies suggest that fermented millet beverages may offer additional benefits, such as reducing cholesterol levels, promoting heart health, and boosting the immune system (Math et al., 2024)	Research in these areas is preliminary, and more studies are needed to confirm these potential benefits.		Paschapur et al., 2021; Sabuz et al., 2023

## 6 Microbial communities in millet fermentation

Recent advancements in microbiology have identified yeasts, bacteria, and molds as the primary microorganisms in millet fermentation. Typically, fermented drinks involve these three groups. The fermentation of cereal-based alcoholic beverages consists of two fundamental processes: (a) enzymatic breakdown and (b) fungal hydrolysis of starch to glucose, followed by yeast fermentation of glucose into ethanol. Notable ethanol-producing yeasts include *Meyerozyma guilliermondii*, *Wickerhamomyces ciferrii*, *Candida glabrata*, *Debaryomyces hansenii*, *Ogataea parapolymorpha*, and *Dekkera bruxellensis*. The microbial community in Xaj starter cakes also features amylase-producing molds such as *Rhizopus delemar*, *Mucor circinelloides*, and various *Aspergillus* species, with lactic acid bacteria (LAB) typically dominating the bacterial community. Specific yeasts generate complex compounds, including fuel alcohols, glycosides, acids, and esters, which contribute to the final products' flavor, aroma, and sensory qualities (Fuloria et al., 2022). The microbes that initiate fermentation are attracting interest because of their recent associations with several health advantages. Using enzymes like proteinase and peptidase, these microorganisms synthesize biologically active peptides, mineral and vitamin synthesis, and some of the non-nutrients being eliminated during fermentation. Consequently, it is also well acknowledged that physiologically active peptides—produced through the bacteria that cause fermentation—have positive benefits. (Table 4) (Sanlier et al., 2019).

Microbes involved in fermentation can withstand the effects of antibiotics. The antibiotic susceptibility of several LAB isolates was investigated in an experiment using various antibiotics. According to the findings, every microbe isolate was resistant to ciprofloxacin, vancomycin, streptomycin, kanamycin, and ampicillin, although different strains and species were sensitive to different amounts of these antibiotics. Microbes are able to withstand antibiotics and survive for a long time in the gut. The fermented cowpea-derived Lactobacillus strain exhibited a high tolerance to antibiotics. (Malongane & Berejena, 2024).

In 2023, LAB such as *Fructilactobacillus sanfranciscensis* I4 and *Companilactobacillus paralimentarius* Fr L19 were utilized, both known for their anti-inflammatory and antioxidant properties, and isolated from naturally fermenting einkorn (Balli et al., 2023).

Most fermented alcoholic beverages are produced through industrial or spontaneous fermentation processes. Dominant microbial communities vary across traditional fermentation methods, including molds like *Aspergillus aceti* and *Rhizopus stolonifer*, LAB species like *Limosilactobacillus fermentum* and *Lactiplantibacillus plantarum*, and yeasts such as *Saccharomyces cerevisiae* and *Candida mycoderma*. Other prevalent genera include *Acetobacter*, *Pseudomonas*, *Klebsiella*, *Weissella*, *Achromobacter*, *Flavobacterium*, *Micrococcus*, and *Bacillus*. The involvement of LAB enhances the shelf life and safety of the finished products (Madilo, Parry-Hanson Kunadu, Tano-Debrah, Saalia, & Kolanisi, 2024). Table 5 lists the key microorganisms, including lactic acid bacteria (LAB), yeasts, and molds, involved in millet fermentation. It describes their specific roles in the fermentation process, such as lactic acid production, flavor enhancement, and nutrient modification, and provides examples of millet-based products resulting from these microbial activities.

**Table 5:** Microorganisms Involved in Millet Fermentation: Genera, Roles, and Final Product Examples.

**Micro-organism Type**	**Dominant Genera**	**Role in Fermentation**	**Specific Examples**	**Final Product Examples**	**Ref.**
**Bacteria (Lactic Acid Bacteria - LAB)**	*Lactobacillus* (plantarum, fermentum, casei)	Produce lactic acid, which lowers pH and preserves product	*Lactobacillus plantarum*, *Lactobacillus paracasei*	Sourdough millet bread, injera, ogi	Ilango & Antony, 2021
	*Pediococcus* (acidilactici, pentosaceus)	Contribute to lactic acid production and aroma development	*Pediococcus pentosaceus*, *Pediococcus acidilactici*	Ben-saalga, hausa koko, burukutu	Bumbie et al., 2024
	*Weissella* (cibaria, confusa)	Produce lactic acid and contribute to flavor and nutritional profile	*Weissella cibaria*, *Weissella confusa*	Millet porridges, fermented millet flours	Compaore-Sereme et al., 2023
	*Streptococcus* (thermophilus)	*May be present in some fermentations, contribute to lactic acid production and aroma*	*Streptococcus thermophilus*	Some millet yogurts and cheeses	Chen & Wang, 2022
**Yeasts**	*Saccharomyces cerevisiae*	*Ferment sugars to produce alcohol (ethanol) and CO2, contributing to flavor and texture*	*Saccharomyces cerevisiae*	Millet beers, wines, and sourdough breads	Balli et al., 2023
	*Saccharomyces boulardii*	*May be used for probiotic benefits and to improve shelf life*	*Saccharomyces boulardii*	Fermented millet flours and porridges	Balli et al., 2023
	*Candida krusei*	*Can contribute to aroma and flavor development in some products*	*Candida krusei*	Some millet beers and wines	Banwo, Asogwa, Ogunremi, Adesulu-Dahunsi, & Sanni, 2021
	*Kluyveromyces marxianus*	*Can contribute to alcohol production and flavor development*	*Kluyveromyces marxianus*	Some millet beers and wines	Atter et al., 2021
**Molds**	*Rhizopus oryzae*	*Used in some Asian-style fermentations to break down starches and contribute to flavor*	*Rhizopus oryzae*	Chinese Jiuhuang wine	Purewal et al., 2020
	*Aspergillus oryzae*	*Used in some fermentations to produce enzymes that break down starches and proteins*	*Aspergillus oryzae*	Some millet miso and shoyu	Purewal et al., 2020

## 7 Critical factors affecting millet fermentation

The primary determinants of fermented food are the microbes that cause fermentation and the chemical structure of the substrates utilized. Food fermentation is also influenced by food treatment and the duration of fermentation throughout preparation. When food deteriorates during regulated or uncontrolled fermentation, the concentration typically rises to the point where the formation is negatively impacted by temperature, pH, oxygen, substrate, and water Table 6 (Sharma et al., 2020).

**Table 6:** Factors Influencing Microbial Dynamics in Millet Fermentation (Cui et al., 2024; Hajar et al., 2024; Kitessa et al., 2024; Math et al., 2024).

**Factor**	**Description**	**Impact on Fermentation**	**Traditional Practices**	**Industrial Practices**
**Temperature**	The range that supports microbial growth (optimal: 30–37 °C).	Affects microbial types, metabolic rates, and bacteriocin production.	Often relies on ambient temperatures, leading to variability.	Controlled using heating/cooling systems for consistency.
**pH**	Critical for LAB growth, typically optimal between 5.0 and 6.0.	Influences growth rates, bacteriocin production, and nutrient solubility.	Utilizes local ingredients for natural pH adjustments.	Monitored and adjusted for optimal conditions.
**Oxygen**	Varies by microbial type; LAB are facultative anaerobes.	Affects metabolic pathways, flavor, and nutritional profile.	Natural methods limit oxygen exposure, enhancing LAB growth.	Advanced gas monitoring and control systems ensure low oxygen levels.
**Water Activity**	Refers to the available moisture for microbial growth (aw).	Critical for preventing spoilage and pathogenic growth; ideal below 0.70 aw.	Managed through salting and drying techniques.	Controlled environmental conditions optimize water activity.
**Substrate**	Source of energy and nutrients; different microbes have varied substrate requirements.	Influences microbial diversity and metabolic processes, affecting flavor and nutrition.	Local grains and legumes enrich substrate diversity.	Substrate optimization through pre-treatment enhances fermentable sugars.

### 7.1 Impact of temperature on millet fermentation

Temperature is a critical factor influencing the microbial dynamics during fermentation, significantly affecting the types of microorganisms that prevail and their metabolic activities. The lowest temperature at which microorganisms cease to grow defines their minimum growth threshold, while the optimal temperature is the range that allows for the most rapid development. Conversely, there is a maximum temperature beyond which bacterial growth becomes inhibited. Understanding these temperature dynamics is essential for optimizing fermentation processes. (Hajar, Novianti, & Harianti, 2024).Research has demonstrated that temperature significantly influences bacteriocin production, a crucial aspect of microbial competition and preservation in fermented products. For example, specific bacteriocins such as lactocin A, enterocin 1146, lactocin S, nisin Z, plantaricin and enterocin 1146 (Bouchefra et al., 2022). Ibrahim et al. (2021) have been found to correlate with optimal growth temperatures, typically ranging from 30 to 37 degrees Celsius. At these temperatures, the metabolic activities of lactic acid bacteria are enhanced, leading to increased bacteriocin production, which can inhibit the growth of spoilage organisms and pathogens. Furthermore, temperature impacts the growth rate of microorganisms, which can influence the fermentation's overall flavor, texture, and nutritional profile. For instance, it has been suggested that lower temperatures may reduce the metabolic rate of L. *amylovorus DCE 471,* subsequently affecting the bacteriocin production due to reduced energy availability for metabolic processes (Costa-Trigo et al., 2020). Most strains capable of producing bacteriocins are typically active within the optimal range of 30 to 37 degrees Celsius, where they can efficiently convert substrates into desirable metabolites (Liang et al., 2025). In both traditional and industrial millet fermentation processes, controlling temperature is vital. In conventional settings, fermentation is often conducted in ambient temperatures, which can vary widely, leading to inconsistent results. However, some artisanal producers manipulate fermentation temperatures using natural insulation methods or monitoring ambient conditions to maintain a more stable environment (Baiano, 2021). In industrial applications, temperature is controlled using modern fermentation tanks equipped with heating or cooling systems to ensure optimal microbial activity, consistency, and product quality.

### 7.2 The role of pH in millet fermentation

The pH of the fermentation medium is another crucial factor, particularly for Lactic Acid Bacteria (LAB), which demonstrates remarkable adaptability to acidic conditions. The optimal pH for bacteriocin production typically ranges from 5.0 to 6.0, which varies depending on the specific bacterial strain. pH affects growth and bacteriocin production and influences cell aggregation, bacteriocin absorption, and proteolytic activity within the fermentation system. The pH of the fermentation medium is a crucial factor that significantly influences the metabolic activity of microorganisms, mainly lactic acid bacteria (LAB). LAB are known for their remarkable acid resistance, allowing them to thrive in the broader pH range compared to many other bacterial species. The culture pH affects LAB's growth rates and plays a pivotal role in bacteriocin production, cell aggregation, and the proteolytic breakdown of proteins (Liang et al., 2025). Understanding the optimal pH for specific microbial strains is essential for maximizing fermentation efficiency and product quality. Research indicates that the ideal pH for bacteriocin production can vary significantly among different LAB strains. For instance, some bacteriocins require a slightly acidic pH range of 5.5 to 6.0 for optimal synthesis, while others are produced more effectively at lower pH levels, typically below 5 (Meade, Slattery, & Garvey, 2020). This variation underscores the importance of selecting appropriate microbial strains based on their specific pH requirements, which can significantly influence fermentation outcomes. At a molecular level, pH affects various biochemical processes, including enzyme activity, nutrient solubility, and microbial cell integrity. For example, a lower pH enhances the solubility of certain nutrients, making them more accessible to LAB, which can subsequently improve their metabolic activity. Additionally, the acidic environment can promote cell aggregation, which enhances the formation of biofilms and contributes to the overall stability of the microbial community during fermentation. In traditional millet fermentation processes, pH control relies on natural environmental variations. Fermenters may use local ingredients that naturally acidify the mixture, such as fruits or fermented plant materials, to create an optimal environment for LAB growth. However, the lack of precise control can lead to inconsistencies in product quality. In contrast, industrial fermentation processes utilize advanced monitoring and control systems to maintain the desired pH throughout fermentation. This precision optimises LAB growth and bacteriocin production, resulting in a more consistent and high-quality product. Furthermore, adjustments to pH can also be made by adding food-grade acids, such as citric or lactic acid, to create an optimal fermentation environment. This practice supports LAB growth and enhances the fermented beverage's overall flavor profile, making it more palatable for consumers (Mujahid et al., 2024).

### 7.3 Oxygen requirements for effective millet fermentation

To encourage or prevent the growth of particular bacteria, the atmosphere or oxygen throughout the fermentation process needs to be controlled and effectively managed. The quantity of oxygen needed by each bacterium varies for fermentation and cell division to occur. (Hajar et al., 2024) The requirements and tolerance of microorganisms to molecular oxygen differ from one another. LABs are facultative anaerobic microbes, meaning they may change an essential component of their metabolic process based on the amount of oxygen in their environment. They can be categorized as homo- and heterolactics attributed to this capacity. Certain bacteria, particularly anaerobic ones, cannot synthesize cytochromes and other heme-containing enzymes. This ability, essential to the electron transport chain, is absent from L. *acidophilus and bifidobacterium species.* The primary energy source for anaerobes is substrate-level phosphorylation, so NAD+ regeneration from NAD is crucial. Oxygen increases the growth level of LAB on glucose, and this effect is amplified in the existence of catalase, which removes H_2_O_2_ from the solution. The finding is that the rate at which ATP is generated from glucose in LAB cultures is one growth-rate limiting parameter (Naghshbandi et al., 2019). Effectively managing atmospheric conditions, particularly oxygen levels, is crucial for optimizing microbial growth and fermentation outcomes in millet fermentation processes. The specific oxygen requirements for different bacteria can vary significantly, influencing their metabolic pathways and overall fermentation efficiency (Hajar et al., 2024). Understanding how to manipulate oxygen levels can encourage the growth of desirable microorganisms while inhibiting less beneficial ones. Lactic acid bacteria (LAB) are classified as facultative anaerobes, which means they can adapt their metabolic processes based on oxygen availability. This adaptability allows them to be categorized into homofermentative and heterofermentative groups. Homofermentative LAB primarily produces lactic acid from glucose, while heterofermentative LAB generates a mix of products, including lactic acid, ethanol, and carbon dioxide (Martínez-Miranda, Chairez, & Durán-Páramo, 2022). The balance between these pathways can significantly influence the fermented millet beverage's flavor, aroma, and nutritional profile. Oxygen levels also affect the synthesis of key enzymes and compounds within microbial cells. For instance, certain bacteria, particularly strict anaerobes, cannot synthesize cytochromes and other heme-containing enzymes essential for the electron transport chain. This deficiency is evident in species such as *Lactobacillus acidophilus* and Bifidobacterium, which rely on substrate-level phosphorylation for energy production. In these anaerobic conditions, the regeneration of NAD+ from NADH is critical for maintaining metabolic activity (Higuchi et al., 2000). In practical terms, oxygen can enhance the growth rate of LAB, mainly when glucose is present. The presence of catalase, an enzyme that detoxifies hydrogen peroxide (H2O2), can further amplify this effect by removing oxidative stress, thereby supporting LAB proliferation and metabolic activity. In traditional millet fermentation processes, oxygen management may rely on natural techniques, such as sealed fermentation vessels or anaerobic fermentation pots, which limit oxygen exposure and create an ideal environment for LAB growth. These methods can lead to the production of more complex flavors and improved nutritional profiles. In industrial settings, oxygen levels are meticulously controlled using advanced fermentation tanks with gas monitoring systems. Techniques such as inert gas flushing or anaerobic chambers can be employed to maintain low oxygen levels, optimizing conditions for the desired LAB strains while inhibiting the growth of spoilage organisms. This precision ensures consistent quality and safety of the final fermented product.

### 7.4 Water activity and its influence on millet fermentation

Without water, microorganisms cannot proliferate. The quantity of water in the medium used for the development of microbes is represented by the ‘water activity (aw)’, which refers to the proportion of the ‘vapour pressure of the solution (P)’ to the ‘vapour pressure of pure water (Po)’ at the same temperature (Hajar et al., 2024). Water activity (aw) is a critical factor in the fermentation of foods, influencing microbial growth and product stability. In the case of reborn shrimp paste, the initial water activity was measured at 0.65 aw, which is conducive to preserving the paste by limiting the growth of spoilage organisms. After 30 days of fermentation, the water activity levels varied depending on the packaging material used, as summarized in Table 1. Notably, using paper, teak leaf, and plastic packaging did not significantly alter the water activity, maintaining it at levels that inhibit microbial spoilage. The structure of the packaging materials plays a crucial role in this context. For instance, while teak leaves have wider surface pores than plastic, they still protect against external contaminants effectively. This characteristic helps maintain low water activity levels, which is essential for preventing pathogenic bacteria and mould growth. Research by Frazier and Westhoff (1988) indicates that maintaining water activity below 0.70 aw is vital to prevent the growth of molds that can compromise food safety. Specifically, pathogenic organisms such as *Staphylococcus aureus* and Salmonella spp. thrive at higher water activity levels, around 0.990–0.995 aw, while *Escherichia coli* requires similar high conditions to flourish. In traditional fermentation practices, managing water activity is often achieved through salting, drying, or using specific fermentation conditions that naturally lower water availability. For example, in shrimp paste production, including salt enhances flavor and reduces water activity, creating an environment where pathogenic bacteria cannot survive. Similarly, during millet fermentation, strategies like controlled drying of millets before fermentation can achieve similar effects, ensuring that the final product remains safe for consumption. Moreover, industrial processes often employ precise control over environmental conditions, such as humidity and temperature, to manage water activity effectively.

### 7.5 Influence of substrate composition on fermentation dynamics

The type of substrate used in millet fermentation is crucial as it directly impacts microbial activity and fermentation outcomes. Food is an energy source providing essential chemical components for microbial growth and development. The biochemical composition of the substrate dictates which microorganisms can thrive during the fermentation process, as different microbes have varying substrate requirements and capabilities to metabolize specific compounds (Hajar et al., 2024). Microorganisms possess particular enzyme systems, or biological catalysts, that allow them to break down substrates into simpler forms. For instance, certain lactic acid bacteria (LAB) are equipped with enzymes that can effectively hydrolyze starches into simpler sugars, making them readily available for fermentation. This ability is essential in millet fermentation, where the starch content of millets can be a significant substrate for LAB. The diversity of substrate requirements among microorganisms means some species thrive on complex carbohydrates while others prefer simpler sugars. For example, while some LAB can metabolize lactose, others may require more basic substrates such as glucose or fructose. This substrate specificity can determine the types of microorganisms that dominate the fermentation process and the final product's resulting flavor, aroma, and nutritional profile. In traditional millet fermentation practices, the selection of substrates often reflects local agricultural practices and food availability. For instance, traditional brews may incorporate various grains and legumes in many African cultures, enriching the substrate diversity and promoting a broader range of microbial interactions. (Taylor et al.,2019). This approach can lead to complex flavor profiles and enhanced nutritional benefits due to the synergistic effects of different substrates.

Moreover, introducing specific adjuncts, such as malted grains or additional sugars, can further influence microbial dynamics. These adjuncts can provide additional nutrients that facilitate the growth of beneficial microbes, thereby enhancing the overall fermentation process (Adebiyi et al.,2018). In industrial settings, substrate optimization is achieved through controlled fermentation processes. This may involve pre-treatment methods such as soaking, grinding, or enzymatic hydrolysis to enhance the availability of fermentable sugars from millet. By carefully selecting and preparing the substrate, manufacturers can promote desirable microbial strains' growth while minimizing spoilage organisms' presence. Table 6 details the critical factors that influence the fermentation process of millet, including temperature, pH, oxygen levels, water activity, and the substrate. It compares traditional and industrial fermentation practices, offering insights into how each factor impacts the quality and consistency of fermented millet products.

## 8 Nutritional and practical advantages of millet fermentation

In addition to improving food quality for individuals who regularly consume millet, preparing dishes and beverages with increased nutritional content using a simple, low-cost process can also encourage cereal to be used more frequently in countries where it is not a staple grain (Table 7). Millet has many nutritional benefits over other popular grains, which are enhanced through fermentation. In foxtail millet fermented with Bacillus natto, there was an increase in fibre content linked to hemicellulose and cellulose breakdown. The fermentation of foxtail millet with *Bacillus natto* increases fibre content due to the enzymatic breakdown of hemicellulose and cellulose, which are significant components of the plant's cell wall. During fermentation, *Bacillus natto* produces specific enzymes, such as cellulases and hemicellulases, that degrade these complex carbohydrates. This process not only breaks down cellulose, a rigid structural carbohydrate made of long glucose chains, but also targets hemicellulose, which consists of various sugar monomers. As these fibres are broken down, they become more soluble and digestible, enhancing the nutritional profile of the millet. The increased fibre content is associated with improved gut health benefits, as the fermentation products can promote the growth of beneficial gut bacteria. Overall, the fermentation process enhances the bioavailability of nutrients and contributes to a healthier food product (Balli et al., 2023).

**Table 7:** Significance of Fermentation in Beverage Production (Bembem et al., 2020; Choudhary, Dhewa, & Kumar, 2024; Fuloria et al., 2022; Panigrahi et al., 2024; Badejo et al., 2020).

**Aspect**	**Significance**	**Examples**
**Flavor Development**	Creates complex flavor profiles through the production of acids, esters, alcohols, and other compounds.	Wine, beer, kombucha, kefir
**Preservation**	Produces natural preservatives, like lactic acid and alcohol, extending shelf life and reducing spoilage.	Pickles, kimchi, vinegar, sauerkraut
**Nutrient Enhancement**	Makes nutrients more bioavailable, increasing overall nutritional value.	Yogurt, kefir, miso
**Digestion**	Introduces live bacteria (probiotics) that aid digestion and improve gut health.	Yogurt, kefir, kombucha
**Beverage Diversity**	Enables the creation of a wide variety of beverages with unique characteristics.	Cider, mead, kvass, tepache
**Sustainability**	Can utilize food waste or locally sourced ingredients, reducing environmental impact.	Kombucha from fruit scraps, beer from spent grains
**Economic Importance**	Supports a multi-billion-dollar industry, creating jobs and promoting cultural heritage.	Wine, beer, coffee, tea
**Innovation**	Opens doors for novel beverage development through experimentation with different microbes and substrates.	Functional beverages with added health benefits, probiotic sodas

The duration of the fermentation process was also crucial in bringing the product's pH within a suitable range [3.89–4.01] and preventing the development of possibly harmful microbes. As the proliferation of lactic acid bacteria (LAB) has led to pH adjustments in the product, aimed at managing the proliferation of Enterobacteriaceae, total coliforms (TC), Staphylococcus species, and aerobic spore-forming bacteria (ASFB) (Kitessa et al., 2024).

Fermentation prolongs shelf life by decreasing pH levels, enhancing acidity, and minimizing microbial contamination. Additionally, it serves a vital function in strengthening sensory attributes such as texture, flavor, and aroma. Furthermore, it lowers the antinutritional components found in cereals while increasing meals' nutritional benefits and digestibility. Additionally, the ‘bio-accessibility’ and ‘bio-availability’ of vital nutrients from many cereal crops are enhanced by fermentation (Table 5) (Alemu & Kuyu, 2024).

Studies have demonstrated that certain types of fermented foods can enhance health by fixing dysbiosis in the gut and encouraging vitamin production, colon health, and immunity against harmful infections. The microorganisms found in naturally fermented African foods have a strong antibacterial activity and a high intestinal adhesion rate (Malongane & Berejena, 2024).

Whether using single or multiple organisms, fermentation of millets generates specific or diverse bioactive peptides and metabolites that support health. This low-cost fermentation process enhances the nutritional value of food and is also considered environmentally friendly. Additionally, millets are utilized to produce distilled beverages that cater to consumers seeking gluten-free and low-calorie options. Their high levels of polysaccharides, phytochemicals, and dietary fibres are beneficial in addressing lifestyle diseases such as diabetes, gastrointestinal disorders, cancer, inflammation, cardiovascular issues, and weight management (Tanwar et al., 2023). Moreover, millets hold potential for the pharmaceutical and nutraceutical industries. Therefore, millet can be regarded as a “savior” in combating malnutrition and lifestyle-related health problems (Math et al.,2024).

In the context of Siddha and Ayurvedic practices, millets have long been recognized for their nutritional and therapeutic benefits, extending to their fermented forms. Fermented millet beverages, such as “kambu koozh” (a traditional South Indian drink made from pearl millet), exemplify this heritage. The fermentation process enhances the bioavailability of nutrients and introduces beneficial probiotics, contributing to gut health and overall wellness (Chaturvedi et al.,2024). Research indicates that these beverages can help regulate blood sugar levels, improve digestion, and provide a rich source of antioxidants. Furthermore, traditional texts emphasize using fermented millets to balance doshas, particularly in promoting digestive fire (Agni) and enhancing metabolic health. The microbial dynamics involved in fermentation play a crucial role in developing these health-promoting properties, highlighting the interplay between traditional dietary practices and modern nutritional science. Incorporating findings from recent studies on the health benefits of fermented millet beverages can further underscore their significance in both Siddha and Ayurvedic traditions (Kulkarni et al., 2023). Table 7 highlights the diverse benefits of fermentation in food and beverage production, from flavor development and preservation to increased nutrient availability and sustainability. It outlines the role of fermentation in enhancing the functional properties of millet-based products and their contribution to sustainability in the food industry. Examples of fermented products are included, alongside their significance in modern food culture and economy.

## 9 Challenges in optimizing millet fermentation processes

### 9.1 Maintaining microbial stability in millet fermentation

Millet fermentation relies on the activity of diverse microorganisms, such as bacteria, yeast, and molds. However, maintaining the stability and balance of microbial communities throughout the fermentation process can be challenging. Unwanted microbial contamination can lead to off-flavors, spoilage, and decreased product quality. Ensuring the dominance of desirable microbes while suppressing the growth of harmful ones is a key challenge in millet fermentation (Ghosh, Bornman, Meskini, & Joghataei, 2024).

### 9.2 Optimizing fermentation conditions for millet beverages

The fermentation process for millet-based products requires precise control of various factors, including temperature, pH, fermentation time, and moisture content. Achieving optimal conditions for microbial growth and metabolite production can be complex, especially in traditional fermentation practices where environmental conditions may fluctuate. Developing robust fermentation protocols tailored to specific millet varieties and product types is essential for consistent quality and reproducibility (Yadav et al., 2024).

### 9.3 Enhancing nutritional profiles of millet beverages through fermentation

While millet is inherently nutritious, fermentation can further increase its nutrition profile by increasing the bioavailability of nutrients and synthesizing beneficial compounds such as vitamins, amino acids, and antioxidants. However, optimizing fermentation conditions to maximize nutritional benefits without compromising sensory attributes and safety remains challenging. Understanding the biochemical transformations occurring during fermentation and their impact on nutritional quality is crucial for developing fortified millet-based products with enhanced health benefits (Hidalgo-Fuentes et al., 2024).

### 9.4 Challenges in scaling up millet fermentation for commercial use

Traditional millet fermentation practices are often small-scale and artisanal, limiting their potential for large-scale production and commercialization. Scaling up fermentation processes while maintaining product consistency, quality, and authenticity poses significant challenges. Additionally, integrating modern technologies and equipment into traditional fermentation practices without sacrificing cultural heritage and authenticity requires careful consideration and adaptation (Mishra et al., 2024).

## 10 Future directions in millet fermentation research

The prospects for fermented millet beverages are up and coming, as ongoing research into the kinetics of probiotic growth during fermentation can significantly enhance commercial applications. Researchers can develop more effective and viable products by examining how probiotics thrive and interact during fermentation and their stability during storage (Meena et al., 2024). This includes investigating innovative techniques, such as the application of ultrasound, which may improve microbial interactions and overall fermentation efficiency. Furthermore, the promotion of millet cultivation offers a sustainable strategy for addressing climate change, given millet's resilience to drought and its adaptability to less fertile soils. Such initiatives not only support agricultural sustainability but also encourage the integration of millets into diverse dietary practices. As we look to the future, there is a compelling need for research to identify optimal combinations of beneficial microbes. This could lead to the development of millet-based foods that are not only nutritious but also tailored to meet specific health needs and consumer preferences (Balli et al., 2023). This multifaceted approach, combining scientific research, sustainable agricultural practices, and market innovation positions fermented millet products as a vital component of future food systems, enhancing their nutritional profile while contributing to food security and environmental resilience.

### 10.1 Advances in microbial ecology and metagenomics for millet fermentation

Advancements in microbial ecology and metagenomic analysis offer exciting opportunities to unravel the complex microbial communities involved in millet fermentation. Studying microbial populations' dynamics, interactions, and functional roles can provide valuable insights into optimizing fermentation processes and controlling microbial spoilage. Additionally, harnessing beneficial microbes through targeted selection and manipulation can enhance fermentation efficiency and product quality (Bhattacharjee et al., 2024). With statistical methods, such as RSM (response surface methodology) and ANN (artificial neural network), significant improvements in bacteriocin output were obtained to optimize culture conditions and medium compositions. These approaches can help fine-tune variables like temperature, pH, and nutrient concentrations, resulting in substantial improvements in producing valuable metabolites, including bacteriocins—compounds with antimicrobial properties that can inhibit spoilage organisms. This research opens avenues for producing higher-quality millet-based products and contributes to food safety and sustainability by minimizing microbial spoilage and maximizing the use of natural fermentation processes (Liang et al., 2025). Overall, these innovations underscore the importance of a comprehensive understanding of microbial ecology in enhancing fermented millet beverages' commercial viability and nutritional profile.

### 10.2 Strain improvement and bioprospecting in millet fermentation

Bioprospecting for novel microbial strains with desirable fermentation traits holds promise for improving the efficiency, flavor, and nutritional quality of millet-based products. Screening microbial isolates from diverse ecological niches for their fermentation capabilities and stress tolerance can lead to the discovery of novel starter cultures and probiotic strains. Furthermore, employing genetic and metabolic engineering techniques to enhance the performance of selected strains offers avenues for tailored fermentation solutions (Cui et al., 2024). This process includes screening for strains that exhibit robust fermentation traits and those that demonstrate resilience under stress conditions, such as high temperatures or low pH, which are critical for successful fermentation. Identifying novel starter cultures and probiotic strains can lead to products with improved health benefits and sensory qualities.

Furthermore, leveraging genetic and metabolic engineering techniques allows researchers to optimize these selected strains for specific fermentation needs, such as enhancing flavor profiles or increasing the production of beneficial metabolites. For example, through targeted genetic modifications, strains can be engineered to produce higher levels of desirable compounds, such as vitamins and organic acids, which contribute to the overall nutritional value of the final product. This tailored approach not only boosts the quality and consistency of millet-based foods but also supports the growing demand for innovative, health-oriented products (Cui et al., 2024). Overall, bioprospecting and strain improvement represent vital components of a broader effort to harness microbial diversity for sustainable and enhanced fermentation practices.

### 10.3 Innovations in Technology for Optimizing Millet Fermentation

Integrating advanced technologies such as bioreactors, omics-based approaches, and artificial intelligence into millet fermentation processes can revolutionize production efficiency, quality control, and product innovation. Automated monitoring systems for real-time process monitoring, predictive modelling for optimizing fermentation parameters, and precision fermentation for customized product development are areas ripe for exploration. Additionally, sustainable and eco-friendly practices such as solid-state fermentation and co-culturing can minimize environmental impact and resource usage (Mishra et al., 2024). Moreover, the application of omics-based approaches—encompassing genomics, proteomics, and metabolomics—provides a comprehensive understanding of the biochemical pathways involved in fermentation. This insight facilitates the identification of key metabolites and microbial interactions, paving the way for targeted enhancements in flavor, texture, and nutritional content.

Artificial intelligence (AI) also plays a transformative role in this context by enabling predictive modelling, which can forecast fermentation outcomes based on various input parameters. This capability not only streamlines the development of new products but also allows for precision fermentation, where specific microbial strains are selected and cultivated to create customized products tailored to consumer preferences. Furthermore, embracing sustainable practices such as solid-state fermentation and co-culturing—where multiple microbial species are fermented together—can reduce resource consumption and environmental impact. Solid-state fermentation often requires less water and energy than traditional methods, while co-culturing can enhance microbial diversity, leading to more complex flavors and improved nutritional profiles. Together, these technological innovations represent a forward-thinking approach to millet fermentation, promoting efficiency and sustainability while meeting the evolving demands of consumers (Mishra et al., 2024).

### 10.4 Expanding millet fermentation markets through diversification

Expanding the market reach of millet-based fermented products through product diversification and value-addition strategies can enhance consumer acceptance and economic viability. Developing novel product formulations, incorporating functional ingredients, and exploring niche markets can create new opportunities for growth and differentiation. Furthermore, promoting millet fermentation's cultural heritage and nutritional benefits through marketing initiatives and consumer education can increase awareness and demand for these products (Mahajan, Singla, & Sharma, 2024). Exploring niche markets, such as gluten-free, vegan, or organic segments, can create significant opportunities for growth and differentiation, as these consumers are often willing to pay a premium for products that align with their values and dietary restrictions. To further support market expansion, marketing initiatives should emphasize millet's cultural heritage and traditional uses, educating consumers about its rich history and nutritional benefits. By highlighting the sustainability and resilience of millet as a crop, companies can tap into the growing consumer interest in eco-friendly and ethically produced foods. Engaging in community outreach, workshops, and tasting events can also foster a deeper connection with consumers, helping to demystify millet and showcase its versatility. Overall, these strategies promote millet-based fermented products and contribute to a broader cultural and nutritional appreciation, driving demand and establishing millet as a staple in modern diets (Mahajan et al., 2024).

## 11 Conclusion

Fermented food and beverages have been a crucial part of human diets for thousands of years due to natural form modifications contributing to high-profile nutritional qualities and better flavor. However, little is understood about bacteria's role in these modifications. By studying the changes in fermented foods that may affect cognition, this work seeks to give a comprehensive knowledge of the positive impacts of bacteria on fermented foods. It accomplishes this by providing a thorough synopsis of fermented foods' biochemical and microbiological alterations. As a result, various health benefits are associated with fermented foods, such as ‘anti-inflammatory’, ‘anti-microbial’, ‘anti-fungal’, ‘antioxidant’, and ‘anti-atherosclerotic’ qualities. Temperature, pH, aeration, substrate concentration, and nutrient availability are some variables that affect fermentation and other metabolic activities. Future developments ought to concentrate on the industrialized nations' consumption of millet, which has the potential to accelerate the industrial revolution.

## Funding

No funds or grants are received.

## Ethical approval

Not applicable.

Consent of participate.

Not applicable.

Consent to publish.

Not applicable.

## CRediT authorship contribution statement

**Tanu Tomar:** Writing – original draft, Resources, Methodology, Formal analysis, Data curation. **Angel Sachdeva:** Writing – review & editing, Visualization, Validation, Resources, Methodology, Formal analysis. **Joydeep Dutta:** Writing – review & editing, Visualization, Resources, Methodology, Formal analysis, Data curation. **Abdel Rahman Mohammad Al Tawaha:** Writing – review & editing, Resources, Investigation, Formal analysis, Data curation. **Arun Karnwal:** Writing – original draft, Visualization, Validation, Supervision, Methodology, Conceptualization. **Tabarak Malik:** Writing – original draft, Resources, Investigation, Formal analysis, Conceptualization. **Manickam Selvaraj:** Writing – review & editing, Visualization, Validation, Resources, Investigation, Data curation.

## Declaration of competing interest

The authors declare that they have no known competing financial interests or personal relationships that could have appeared to influence the work reported in this paper.

## Data Availability

No data was used for the research described in the article.
